# Application of an Integrative Drug Safety Model for Detection of Adverse Drug Events Associated With Inhibition of Glutathione Peroxidase 1 in Chronic Obstructive Pulmonary Disease

**DOI:** 10.1007/s11095-023-03516-x

**Published:** 2023-05-12

**Authors:** Jack L. Janetzki, Nicole L. Pratt, Michael B. Ward, Matthew J. Sykes

**Affiliations:** 1 UniSA: Clinical and Health Sciences, University of South Australia, GPO Box 2471, Adelaide, South Australia 5001 Australia; 2 Quality Use of Medicines and Pharmacy Research Centre, Clinical and Health Sciences, University of South Australia, GPO Box 2471, Adelaide, SA 5001 Australia

**Keywords:** chronic obstructive pulmonary disease, glutathione peroxidase, molecular docking, pharmacoepidemiology, pharmacovigilance

## Abstract

**Background:**

Chronic Obstructive Pulmonary Disease is characterised by declining lung function and a greater oxidative stress burden due to reduced activity of antioxidant enzymes such as Glutathione Peroxidase 1.

**Objectives:**

The extent to which drugs may contribute to this compromised activity is largely unknown. An integrative drug safety model explores inhibition of Glutathione Peroxidase 1 by drugs and their association with chronic obstructive pulmonary disease adverse drug events.

**Methods:**

*In silico* molecular modelling approaches were utilised to predict the interactions that drugs have within the active site of Glutathione Peroxidase 1 in both human and bovine models. Similarities of chemical features between approved drugs and the known inhibitor tiopronin were also investigated. Subsequently the Food and Drug Administration Adverse Event System was searched to uncover adverse drug event signals associated with chronic obstructive pulmonary disease.

**Results:**

Statistical and molecular modelling analyses confirmed that the use of several registered drugs, including acetylsalicylic acid and atenolol may be associated with inhibition of Glutathione Peroxidase 1 and chronic obstructive pulmonary disease.

**Conclusion:**

The integration of molecular modelling and pharmacoepidemological data has the potential to advance drug safety science. Ongoing review of medication use and further pharmacoepidemiological and biological analyses are warranted to ensure appropriate use is recommended.

**Graphical Abstract:**

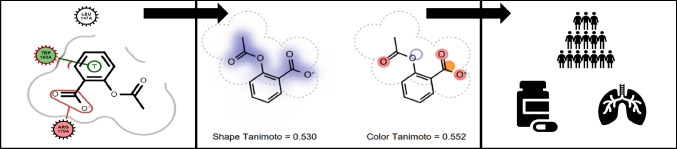

**Supplementary Information:**

The online version contains supplementary material available at 10.1007/s11095-023-03516-x.

## Introduction

Adverse Drug Events (ADEs) are undesirable outcomes that occur following administration of a drug regardless of whether causality has been determined [1]. Pharmacovigilance incorporates the prediction and review of ADEs based on reviewing large data repositories and predicting the potential for ADEs based on proposed pharmacological mechanisms [2, 3].

For example, epidemiological studies have reported that co-administration of certain medicines may increase or reduce the risk of Chronic Obstructive Pulmonary Disease (COPD). Consumption of statins was associated with a reduced risk of COPD whereas consumption of paracetamol (acetaminophen) was associated with an increased risk of COPD [4, 5]. At a pharmacological level, it is largely unknown to what extent medicines impact the activity of antioxidative enzymes such as Glutathione Peroxidase 1 (GPx1) which are involved in the pathogenesis of COPD [5–7].

COPD is an umbrella term for progressive respiratory conditions such as emphysema, chronic bronchitis and chronic asthma which are characterised by inflammation and fibrosis of the airways [8]. COPD is insidious and often occurs due to frequent and prolonged exposure to environmental factors such as cigarette smoke, air pollutants and occupational dusts that increase oxidative stress on cells in the human body [7]. Oxidants in these pollutants may produce reactive oxygen species (ROS) on exposure to the lungs causing damage and inflammation [9]. The lungs have several antioxidant enzymes that exist to reduce damage caused by ROS including catalase, superoxide dismutase and glutathione peroxidase (EC 1.11.1.9). GPx1 is a ubiquitous enzyme which is involved in detoxifying hydrogen peroxide to water and lipid peroxides to alcohols [7]. GPx1 achieves its function by using two molecules of glutathione as cofactors which are ultimately reduced to glutathione disulfide in a two-step process [10]. The active site selenocysteine residue is important in this process. First, upon encountering the hydrogen peroxide the selenol is oxidized to selenenic acid. Then a glutathiolated selenol intermediate is formed after reduction of the selenenic acid by the first glutathione molecule. Subsequently, reduction of the glutathiolated selenol bond occurs by a second molecule of glutathione, restoring the active site and formation of oxidised glutathione disulfide [10].

Erythrocyte activity of GPx1 is reduced in individuals who smoke cigarettes and in those living with COPD. However, GPx1 genes are also upregulated in the same populations [11–13]. This demonstrates that the protective effects of GPx1 against inflammation and damage induced by ROS is reduced in the presence of the increased oxidative stress burden which occurs in COPD [10].

Historically*, in silico* molecular modelling has been utilised to understand the activity of drugs at targets of interest [14, 15]. Changes in the chemical structure of drugs are sometimes subtle and can affect the clinical risk profile. For example, the tyrosine kinase inhibitor sunitinib has been associated with risk of hypothyroidism, however, sorafenib of the same class does not appear to be associated with this risk [14]. *In silico* molecular modelling analyses have demonstrated that sunitinib is more likely to bind to retinoic acid receptors than sorafenib and contribute to dysregulation of the thyroid hormone [14]. Similarly, using molecular docking simulations, paracetamol has been shown to competitively inhibit the binding of carbamazepine to HLA-B*15:02 which reduces the likelihood of severe and potentially fatal cutaneous reactions [16]. This competitive inhibition is likely due to the structural similarity of amino ketone groups and aromatic rings between carbamazepine and paracetamol [16].

Herein we utilise an integrative approach to investigate the potential for Food and Drug Administration (FDA) approved drugs to inhibit GPx1 and association with COPD ADEs. Such integrative approaches have been suggested yet are not widely utilised [17, 18]. Initially, ligand- and structure-based *in silico* modelling techniques will be used to predict the potential for drugs to interact with GPx1. Metrics derived from these models will then be applied to select leading candidates of interest for investigation using pharmacoepidemiologic data sources. Specifically, the Food and Drug Administration Adverse Event Reporting System (FAERS) was searched for ADE reports between drugs suggested by the *in silico* modelling and outcomes of interest in order to investigate real-world evidence of the potential ADE and increased risk of COPD [19].

## Materials and Methods

### Database Curation

A database of 2381 FDA approved drugs was obtained from DrugBank (Approved Drugs, Version 5.1.1, released 3^rd^ July 2018) in Simplified Molecular-Input Line-Entry System (SMILES) format [20, 21]. In addition, the SMILES of several known inhibitors of GPx1 previously confirmed to have GPx1 inhibitory activity were obtained from the literature to represent positive controls for the *in silico* analysis and added to the larger database (see Table I).

**Table I:** Known inhibitors of Glutathione Peroxidase

Molecule name in literature	Chemical name	Chemical structure	GPx inhibitory activity
Compound 3	N-[(E)-(4-hydroxyphenyl)methyleneamino]-2-(2-methylimidazol-1-yl)acetamide		15% inhibition at 50μM IC50= 169 ± 39μM [22]
Mercaptosuccinate	2-sulfanylbutanedioate		0% GPx activity at 0.2mM [23]
3aC7	N-[1-(4-hydroxyphenyl)ethylidene]-2-(1H-imidazol-1-yl) acetohydrazide		47.1 ± 11.6 GPx activity at 80μM [24]
3aC9	*N* ′*-[1-(4-hydroxy-3-methoxyphenyl)ethylidene]-2-(1H-imidazol-1-yl)acetohydrazide*		84.9 ± 7.1 GPx activity at 100μM [24]
3eC7	*N* ′*-[1-(4-hydroxyphenyl)ethylidene]-2-(1H-pyrazol-1-yl)acetohydrazide*		82.7 ± 13.0 GPx activity at 100μM [24]
3bC9	*N* ′ *-[1-(4-hydroxy-3-methoxyphenyl)ethylidene]-3-(1H-imidazol-1-yl)propiohydrazide*		80.2 ± 7.0 GPx activity at 100μM [24]
Misonidazole	(2S)-1-methoxy-3-(2-nitroimidazol-1-yl)propan-2-ol		0.5mM of misonidazole causes 60-70% depression of GPx activity compared to control activity [25]
Tiopronin	2-[[(2S)-2-sulfanylpropanoyl]amino]acetate		60 ± 3% GPx activity at 200μM [23, 26] GPx IC50=365 μM [27]
β-mercaptovaline	(2R)-2-azaniumyl-3-methyl-3-sulfanyl-butanoate		0.5% mean GPx activity at 0.2mM [23]
Demethyltiopronin	2-[(2-sulfanylacetyl)amino]acetate		54 ± 1% GPx activity at 200μM [26]

All *in silico* analyses were performed using the OpenEye Scientific Software molecular modelling software suite [28].

All molecules from the FDA approved drugs DrugBank subset and known inhibitors were subjected to the Openeye Blockbuster filter (part of the OMEGA suite version 4.1.2.0) to remove non-druglike compounds and limit the production of spurious hits [29]. After this filter was applied to the dataset, 1500 molecules (including all of the known inhibitors listed in Table I) remained in the dataset. A maximum of 100 conformers were then generated for each molecule using OMEGA. The limit of 100 conformers was chosen as this is considered to be a standard number of conformers to be used in molecular modelling analyses and to balance computational time requirements whilst ensuring the production of an appropriate ensemble size [29]. Stereochemistry was retained for molecules for which it was already identified. Where stereochemistry was not pre-identified, conformers were generated with the flag ‘strictstereo false’. All other OMEGA settings were used as default. Following conformer generation 1494 molecules remained in the final dataset which was utilised for all subsequent analyses.

### Structure-Based Analyses

Two crystal structures of GPx1 were obtained from the RCSB Protein Databank (PDB); bovine erythrocyte GPx1 (PDB Code: 1GP1) and human erythrocyte GPx1 (PDB Code: 2F8A) [30-32]. For each crystal structure, the active site was modelled using Make Receptor (version 3.4.0.2). The 1GP1 active site model was curated by identifying key amino acids that surround the active site known to have roles in catalysis and substrate or inhibitor binding (GLN140 (glutamine), ARG177 (arginine), SEC45 (selenocysteine) and ARG50 (arginine)), as no bound ligand was present [22, 33–38]. The selenocysteine residue in the active site was also adapted to its reduced form. A box was then determined (dimensions 21.33 Å × 15.00 Å × 10.00 Å) to encapsulate the topography of the key residues of the active site, approximately centered on ARG177. The molecular cavity detection algorithm within Make Receptor was then used to define the active site shape and size, which was determined to have a volume of 1405 Å^3^. As no co-crystallised ligand was present in the PDB 1GP1 entry, the co-factor glutathione was re-docked into the receptor created by Make Receptor to verify the model and ensure that the cysteinyl sulfur of glutathione was oriented to and forming an interaction with the selenocysteine. The known inhibitor tiopronin (glutathione analogue) was also investigated in a similar way and was observed to have the expected orientation within the active site.

The 2F8A binding site was created in a similar way to that of 1GP1 to determine the active site box (dimensions 16.33 Å × 10.67 Å × 14.00 Å). The molecular cavity detection algorithm within Make Receptor was then used to define the active site shape and size, which was determined to have a volume of 1378 Å^3^. The active site selenocysteine in the 2F8A GPx1 crystal structure was found to be mutated to glycine (GLY47) [31]. According to the Uniprot Alignment, there is 87.4% amino acid sequence similarity between bovine and human erythrocyte GPx1 [39].

Docking studies were undertaken on the 1494 molecule conformational database using the FRED algorithm within the OEDocking suite [34]. Docking scores were obtained for each molecule that could be successfully docked into the active site of GPx1, and molecules were subsequently ranked based on their Chemgauss4 docking score [33].Only molecules that ranked within the top 150 docking scores (top 10%) for each model (1GP1 or 2F8A) were considered for subsequent pharmacoepidemiological analyses as these drugs were more likely to potently bind to the target and exert their effects [40–43]. Docking results were visualized in VIDA (version 4.4.0.4) allowing for observations to be made on relative docking ranks and identification of key intermolecular interactions. Protein–ligand interactions were further investigated using the Open Eye Scientific Grapheme Tool Kit 2017 June release (complex2img.py).

### Ligand-Based Analyses

Using the 1494 molecule conformational database, ligand-based modelling was conducted using Rapid Overlay of Chemical Structures ((ROCS) version 3.3.2.2)) [44]. Chemical similarity was measured between tiopronin (the query molecule), a known inhibitor of GPx1 and registered drug, and all other molecules in the dataset. The similarity between tiopronin and each database molecule was assessed and ranked using the Tanimoto Combo Score (linear combination of the Shape and Color Tanimoto values).

### Pharmacoepidemiological Analyses

The Food and Drug Administration (FDA) Adverse Event Reporting System (FAERS) was utilised to search for reports that included medicines for which there was a potential adverse event signal related to COPD or deterioration in lung function [19]. SAS statistical software version 9.4 was used for all FAERS data analysis.

#### Linkage of FAERS Data Files

**Fig. 1:**
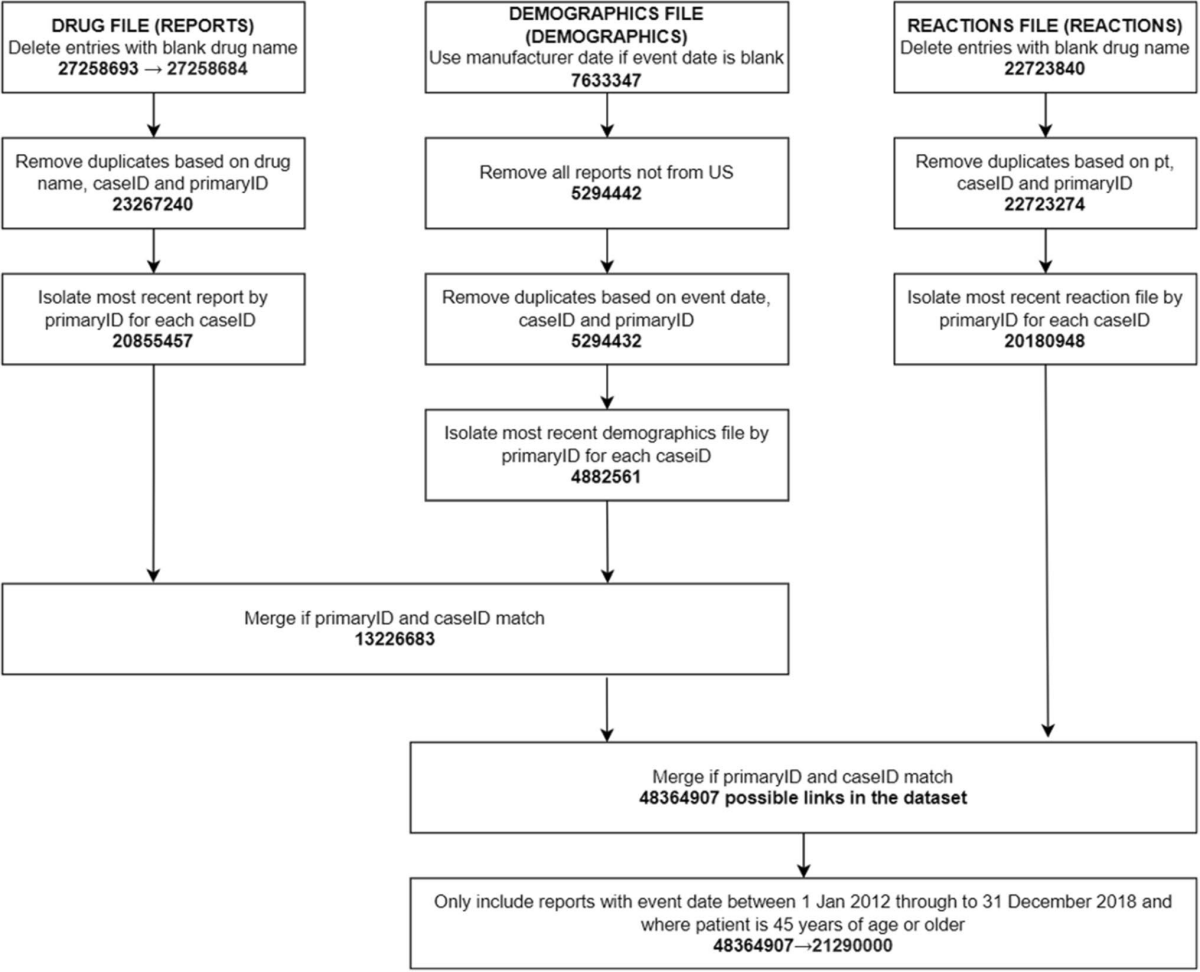
Process of linking and optimising FAERS dataset prior to analysis.

FAERS datasets were linked as described in Fig. 1.

As a FAERS case (CaseID) may contain multiple updated versions of the report (primaryID), only the most current report for each case was kept as this was the most complete and accurate report available [45]. Where the event date of the report was left blank the manufacturer report date was utilised to ensure that a study period could be defined. Each reaction was linked to each drug in the report to remove reporter biases which include whether the reporter suspected the medications caused a particular adverse event. Only reports from the US were captured to ensure drug names and trade names were standardised. Following this filtering process there were approximately 48.4 million possible drug-reaction links in the dataset (Fig. 1). Reports were further limited to those with an event date from 1^st^ January 2012 through to 31^st^ of December 2018 and where the patient was 45 years or older as there is low incidence of COPD before 45 years of age and low likehood of patients developing symptoms of COPD before 45 years of age [46, 47]. The final remaining number of drug-reaction associations was approximately 21.3 million.

#### Implementation of Method on Dataset

Any drug molecule that was ranked in the top 150 in either the 1GP1 or 2F8A models in the structure-based analyses or in the top 150 rank of the ligand-based screening against tiopronin was made available for inclusion as a drug of interest in the FAERS analysis (top 150 results for each method found in Supplementary Tables 1 and 2). Drugs were then included in the FAERS analysis if at least one ‘Trade Name’ was present in the drug monograph on Micromedex [48]. All available generic and trade names available were included in the regular expression search terms when searching FAERS. Regular expressions were also used to identify the drug reaction of interest (categorised by “preferred term” in FAERS) [49]. Reactions of interest included “dyspnoea”, “asthma”, “chronic obstructive pulmonary disease” (COPD), “obstructive airways disease” (OAD), “wheezing”, “cough”, “bronchospasm”, “asthma-chronic obstructive pulmonary disease”, “alveolar lung disease”, “emphysema”, “combined pulmonary fibrosis and emphysema”, “idiopathic pulmonary fibrosis” (IPF), “irregular breathing”, “respiration abnormal”, “productive cough” or “respiratory disorder”.

Reporting odds ratios (ROR) were determined for each adverse event and drug molecule pair as described previously elsewhere [50, 51]. RORs with at least 3 cases and where the lower 95% confidence interval value was greater than 1 were retained for further investigation as this indicated a statistically significant adverse disproportionality signal of potential clinical interest [52–54].

## Results and Discussion

### Multi-Method Model Results Summary

In the structure-based analyses utilising the 1GP1 crystal structure of GPx1, 99.9% (1492/1494) of molecules were successfully docked into the crystal structure. Digoxin and digitoxin were not successfully docked into 1GP1. Similarly, 99.7% (1490/1494) of molecules were able to be docked into the active site of GPx1 crystal structure 2F8A. Delamanid, novobiocin, digitoxin and digoxin were not docked into the active site of 2F8A. The top 150 docking results for structure-based analyses utilising 1GP1 and 2F8A are listed in Supplementary Table 1.

The GPx1 active site is relatively accessible by substrates or co-factors such as glutathione and hosts the unique amino acid selenocysteine [55]. The catalytic triad has previously been identified as selenocysteine(SEC)-glutamine(GLN)-tryptophan(TRP) for Glutathione Peroxidase 1 [56]. Arginine residues (ARG) are also prominent in the active site (ARG50 and ARG177) and may have roles in catalysis and substrate or inhibitor binding [30]. Moreover, glutathione can form interactions with ARG177, GLN140 and SEC45 when it is present in the active site [30]. Only four crystal structures of GPx1 are available in the Protein Data Bank [32]. All were deposited as their apo-crystal structures; none has a bound ligand present. The bovine and human models (1GP1 and 2F8A respectively) were chosen for this study given their mammalian origin and amino acid sequence similarity (87.4%). Moreover, the other two GPx1 crystal structures were of parasitic origin, *Schistosoma mansoni*, and lacked amino acid residues that were important for glutathione binding in the bovine and human models[57]. It was also important to compare results for the 2F8A human model with those of another structure-based model of GPx1 given that the selenocysteine in the active site of 2F8A is mutated to glycine which may affect the binding poses of ligands predicted by the model.

Tiopronin was utilised as the query molecule in the ligand-based model as it has demonstrated therapeutic properties by inhibiting GPx1 activity and affecting cell survival [23, 26]. Tiopronin is structurally similar to the GPx1 cofactor glutathione and was observed to align in a similar orientation to glutathione in the active site of GPx1 in the bovine model. Moreover, in a bovine erythrocyte model, an IC_50_ of 356 µM was reported for tiopronin. In the same study, tiopronin was shown to be cytotoxic to two different human cancer cell lines [27]. Tiopronin is also a registered medication for preventing kidney stones in patients with cystinuria and it shares structural similarity with penicillamine due to the presence of a sulfhydryl group [58]. The top 150 ligand-based modelling results with tiopronin as the query molecule are listed in Supplementary Table 2.

Despite the potential mechanism of GPx1 inhibition contributing to COPD, there is little information describing the potential real-world impact that registered medicines may have on development or exacerbation of COPD. As such, the FAERS database was investigated for adverse drug event reports of COPD with drugs of interest from the molecular modelling analyses.

To be considered for the FAERS analysis, molecules must have appeared in the top 150 in at least one of the structure- or ligand-based models. Table II includes drugs of clinical interest where an adverse event was reported at least 3 times with concomitant administration of the drug of interest, and where the ROR lower 95%CI was greater than 1. For all other adverse events that were searched for each drug, there was either less than 3 adverse event reports or the ROR lower 95% CI was less than 1. Several drugs were unable to be searched because they were not available in the US during the study period or there were no reports in FAERS associated with the use of the drug of interest. Additionally, drugs which are used to treat COPD are not included in this table as confounding by indication is likely to have influenced frequency of reporting in the FAERS database. Molecular modelling ranks (based on Chemgauss 4 for structure-based models and TanimotoCombo for the ROCS ligand-based model) are also provided for each of these molecules of interest in Table II.

**Table II:** Selected molecules of clinical interest with adverse event signals associated with COPD or deterioration in lung function listed in alphabetical order. Entries in the table where more than one adverse event is listed for a drug are ordered by Reporting Odds Ratio lower 95% confidence interval

Drug	Total number of reports in FAERS including this molecule	RORs where ≥ 3 reports and lower 95% CI ≥ 1.			1GP1 Rank	2F8A Rank	Tiopronin ROCS rank
		Adverse event	Number of reports with molecule of interest	Reporting Odds Ratio			
Acetazolamide	2034	COPD	11	2.71 (1.50−4.89)	218	125	62
Acetylcysteine	1904	Wheezing	6	3.05 (1.37−6.80)	196	8	3
		Dyspnoea	57	2.05 (1.57−2.66)			
		Productive cough	8	3.41 (1.70−6.83)			
Acetylsalicylic acid	422311	Dyspnoea	6555	1.05 (1.02−1.07)	169	67	30
Atenolol	79333	Obstructive Airways Disease	15	2.54 (1.53−4.22)	54	400	666
		Irregular breathing	4	5.69 (2.11−15.31)			
Azathioprine	17748	Idiopathic Pulmonary Fibrosis	5	4.09 (1.70−9.84)	254	126	370
Bendamustine	3784	Respiratory disorder	5	3.27 (1.36−7.86)	394	136	440
Cefdinir	4015	COPD	17	2.11 (1.31−3.41)	918	132	716
		Productive cough	14	2.82 (1.67−4.77)			
Dexlansoprazole	18079	Productive cough	32	1.43 (1.01−2.02)	204	111	773
		Cough	137	1.21 (1.02−1.43)			
Epinephrine	14877	COPD	41	1.38 (1.01−1.87)	25	52	133
		Dyspnoea	277	1.26 (1.12−1.41)			
		Asthma	57	2.90 (1.24−3.76)			
		Obstructive Airways Disease	5	4.50 (1.87−10.82)			
		Wheezing	42	2.73 (2.02−3.70)			
		Respiratory disorder	19	3.16 (2.02−4.96)			
		Bronchospasm	16	8.78 (5.37−14.36)			
Furosemide	223695	Respiratory disorder	111	1.23 (1.02−1.48)	144	229	409
		Obstructive Airways Disease	29	1.74 (1.21−2.52)			
		Emphysema	72	1.62 (1.28−2.04)			
		Idiopathic Pulmonary Fibrosis	34	2.23 (1.59−3.13)			
		Dyspnoea	5473	1.67 (1.63−1.71)			
		COPD	795	1.79 (1.67−1.92)			
Labetalol	9150	Respiratory disorder	8	2.16 (1.08−4.33)	41	839	1069
Mycophenolic acid	28858	Obstructive Airways Disease	7	3.25 (1.55−6.83)	101	157	508
Nicotine	25047	COPD	68	1.36 (1.07−1.72)	284	181	94
Pentamidine	1451	Wheezing	7	4.68 (2.23−9.83)	138	985	1240
		Bronchospasm	6	33.75 (15.12−75.32)			
Ribavirin	72863	Dyspnoea	1311	1.22 (1.15−1.28)	126	44	259
Tranexamic acid	434	Obstructive Airways Disease	3	93.08 (29.87−290.06)	71	88	13
Vitamin C	77711	Idiopathic Pulmonary Fibrosis	10	1.87 (1.00−3.48)	102	89	136
Voriconazole	8525	Respiratory disorder	9	2.61 (1.36−5.03)	731	81	562

ARG177 was observed to be one of the most important residues for drug binding in the active site of 1GP1. Interactions with ARG177 included hydrogen bonding (e.g. acetazolamide, acetylsalicylic acid, atenolol, azathioprine, furosemide, mycophenolic acid, ribavirin), cation-pi or ionic interactions. Similarly in 2F8A, ARG179 (the residue which corresponds to ARG177 in the active site of 1GP1) was observed to be similarly important for protein–ligand binding [35]. SEC45 and ARG50 have also been previously observed to be able to form interactions with known inhibitors [10, 22, 26]. No drugs in Table II were observed to form specific interactions with the selenocysteine residue (SEC45) in 1GP1 or with the glycine residue (SEC mutated to GLY47) in 2F8A, however 6 of the 18 drugs (including atenolol (Fig. 3)) were able to form interactions with GLY48 in 2F8A which is located adjacent to GLY47 in the active site. None of the 18 drugs were shown to form specific interactions with ARG50 in 1GP1, however 5 of 18 drugs, including atenolol (Fig. 3) were able to form hydrogen bonds, cation-pi or ionic interactions with the corresponding residue ARG52 in 2F8A. Hydrogen bonds were also commonly formed with ASP135, THR141 and ARG178 in 1GP1 in this analysis indicating that interactions with these amino acids are important for inhibitor binding [22]. In the 2F8A active site, drugs also commonly formed hydrogen bonds, cation-pi, ionic or pi-pi interactions with GLN82 and TRP160 indicating their potential role in inhibitor binding. Selected drugs from Table II (including acetylsalicylic acid, atenolol and nicotine) are discussed as examples in the following section due to their widespread use as therapeutic agents in clinical practice.

### Acetylsalicylic Acid and Atenolol

**Fig. 2:**
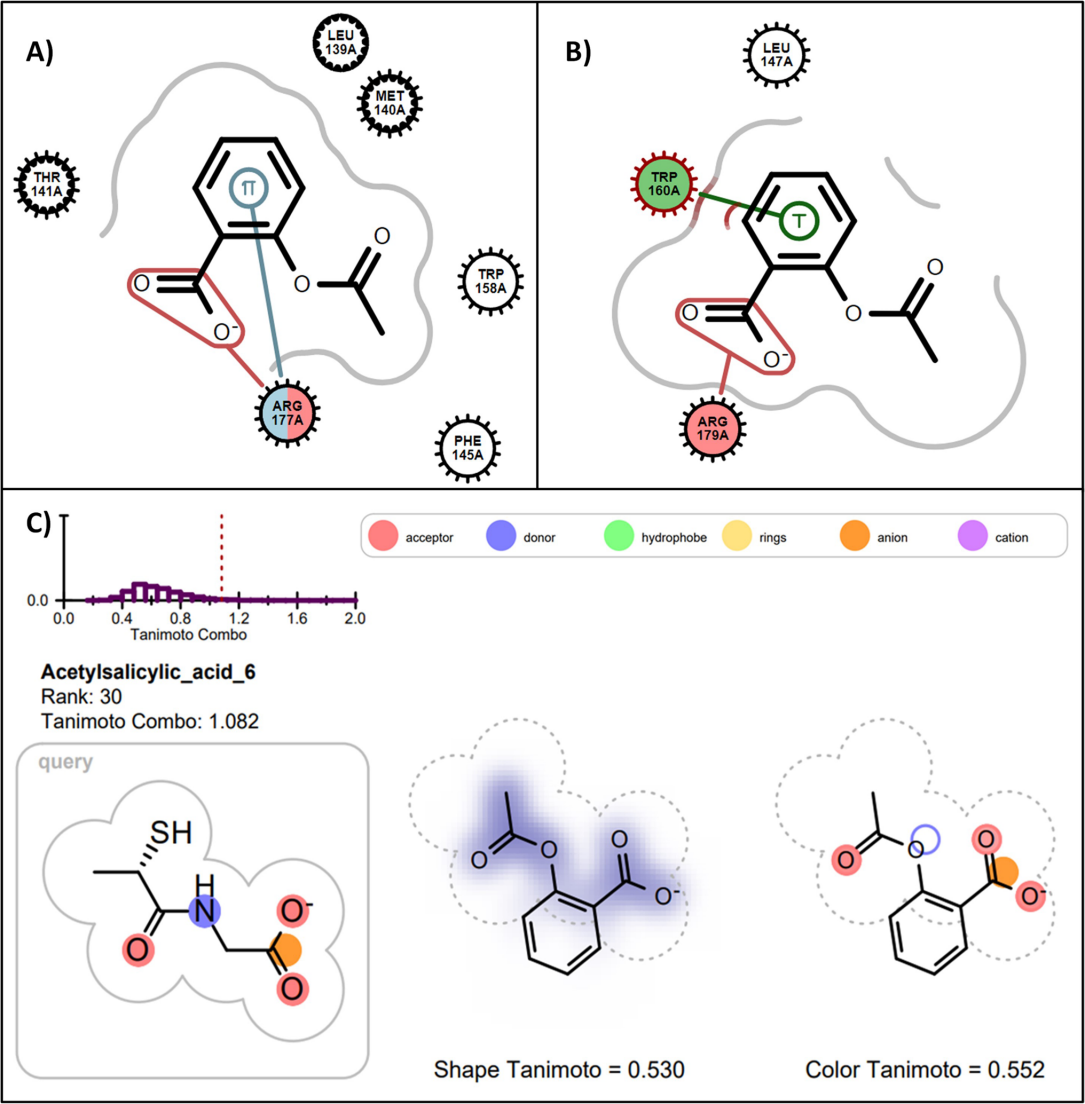
(**A**) Acetylsalicylic acid docked into 1GP1 (rank 169^th^), forming a salt bridge with ARG177 and a cation-pi interaction with ARG177. (**B**) acetylsalicylic acid docked into 2F8A (67^th^), forming an edge-to-face pi stacking interaction with TRP160 and a salt-bridge interaction with ARG179. (**C**) ROCS overlap of acetylsalicylic acid with query molecule tiopronin and its TanimotoCombo rank with respect to all other drugs in the ROCS analyses. Panels A and B were created using the OE Chem Toolkit and Panel C was adapted from the ROCSReport utility program; both part of the OpenEye Software suite.

Acetylsalicylic acid and atenolol (Table II) have been previously shown to contribute to exacerbations of lung conditions by other pharmacological mechanisms which may present differently to GPx1 inhibition [59–64]. However, in this study, acetylsalicylic acid ranks highly in docking in both molecular models of GPx1 (169^th^ in 1GP1 and 67^th^ in 2F8A) indicating that it has the potential to interact with GPx1 and alter its activity. In both models it occupies the Phe145-Trp158-Arg177 region which has previously been shown to be the binding site of glutathione disulfide during the redox reaction of glutathione [35]. Acetylsalicylic acid also ranks highly in the tiopronin ROCS model (30^th^). The ROCS overlay of acetylsalicylic acid and tiopronin in Fig. 2 show that acetylsalicylic acid shares similar chemical features to tiopronin in terms of three hydrogen bond acceptor groups and the anionic carboxylate group. In the FAERS analysis, dyspnoea seems to be reported more frequently for acetylsalicylic acid compared to other drugs (ROR 1.05 95% CI 1.02–1.07). Its use is also associated with acute bronchospasm in approximately 10% of people diagnosed with asthma [65, 66]. Moreover, GPx activity has been shown to be significantly compromised in the red blood cells of patients with acetylsalicylic acid-induced asthma compared to control patients [65]. Further analyses are required to determine whether acetylsalicylic acid alters GPx1 function *in vitro* at either antiplatelet or analgesic doses.

**Fig. 3:**
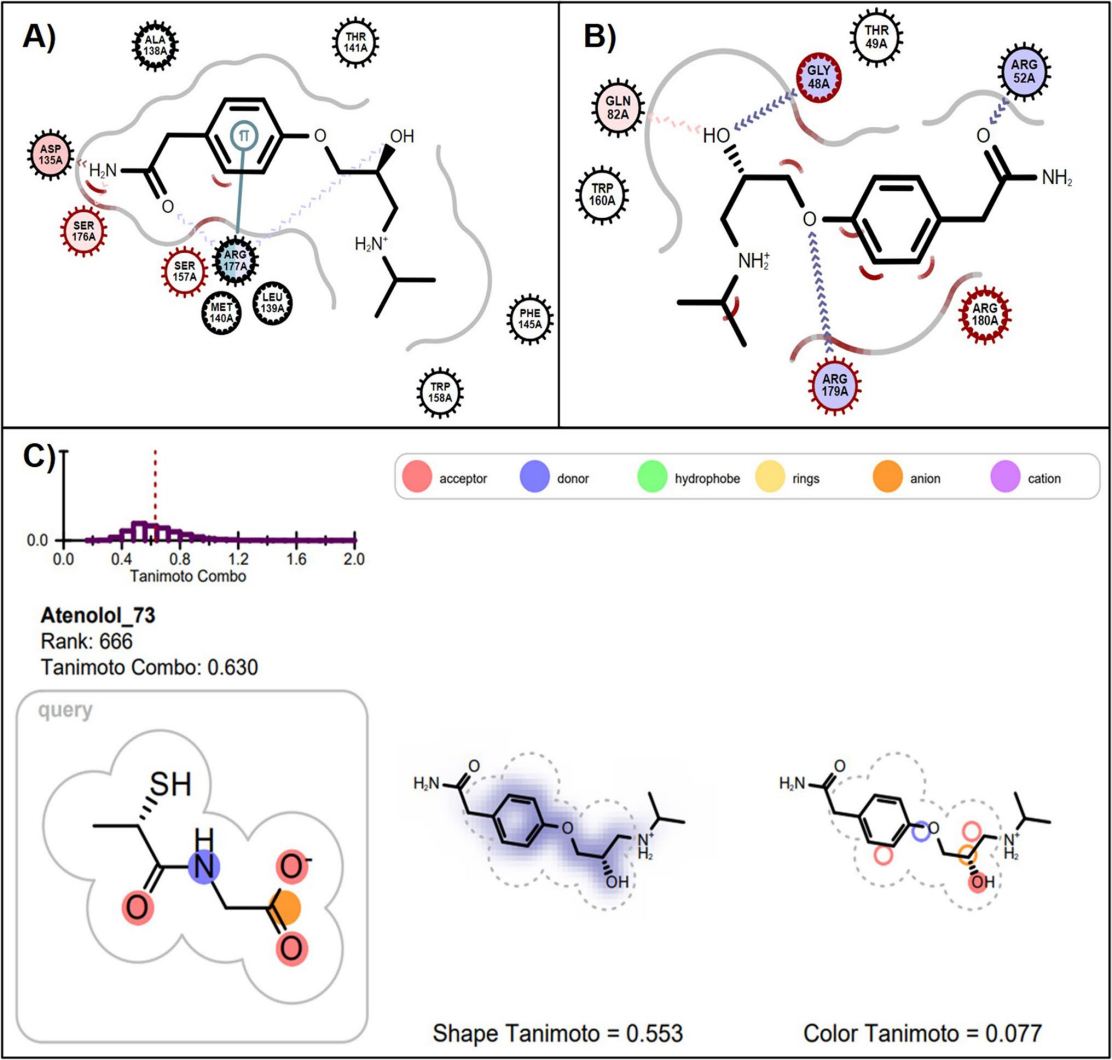
(**A**) Atenolol docked into active site of 1GP1 (ranked 54th) forming one hydrogen bond with ARG177, ASP135 and SER176. Atenolol also has a cation-pi interaction with ARG177. (**B**) Atenolol docked into active site of 2F8A (ranked 400th) forming hydrogen bonds with GLY48, ARG52, GLN82 and ARG179. (**C**) ROCS overlap of atenolol with query molecule tiopronin and its TanimotoCombo rank with respect to all other drugs in the ROCS analyses. Panels A and B were created using the OE Chem Toolkit and Panel C was adapted from the ROCSReport utility program; both part of the OpenEye Software suite.

Atenolol ranks highly in docking into the active site of 1GP1 (54^th^) demonstrating that it may have the potential to alter GPx1 function at a molecular level. In Fig. 3, atenolol was observed to form hydrogen bonds with ARG177, ASP135 and SER176 in the 1GP1 crystal structure of GPx1. Cation-pi interactions were observed between the aromatic ring of atenolol and the ARG177 residue. Additionally, in the 2F8A model, although atenolol docked poorly (400^th^) it was observed to form hydrogen bonds with GLY48 which is close to the GLY47 residue. In the ROCS analysis with tiopronin as the query, atenolol did not rank favourably (666^th^). However, in the FAERS analysis, the cardio-selective beta-blocker atenolol was positively associated with obstructive airways disease (ROR 2.54 95% CI 1.53–4.22) and irregular breathing (ROR 5.69 95%CI 2.11–15.31). Similarly, labetalol, a non-selective beta blocker was associated with respiratory disorder (ROR 2.16 (95%CI 1.08–4.33)) and ranked 41^st^ in the 1GP1 docking analysis. Orally administered labetalol has previously been associated with higher incidence of asthma exacerbations when compared to placebo [67]. Debate continues on the safety of beta-blockers and respiratory conditions [61, 68].

### Nicotine

**Fig. 4:**
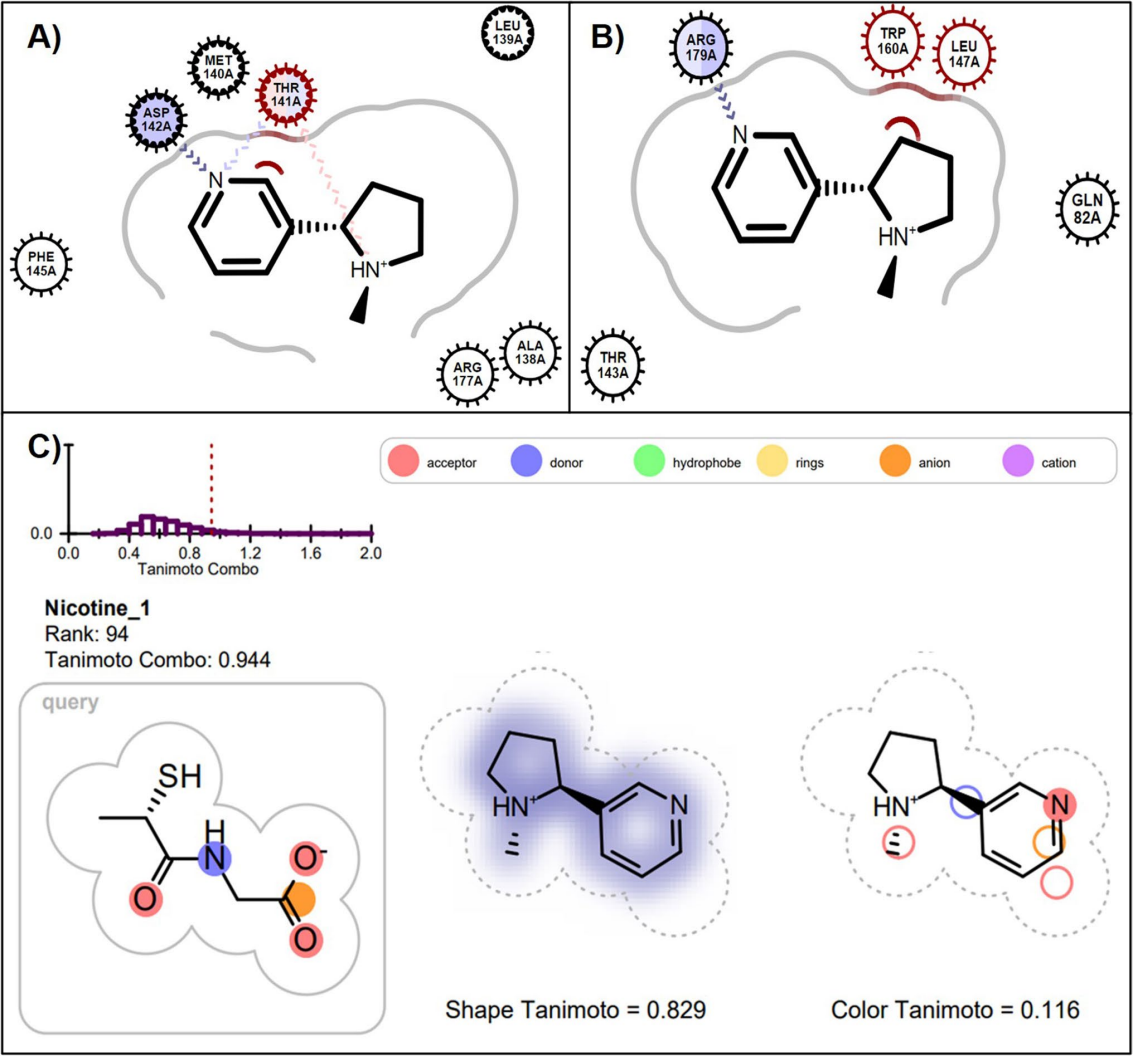
(**A**) Nicotine docked into active site of 1GP1 (ranked 284th) forming two hydrogen bonds with THR141 and another with ASP142. (**B**) Nicotine docked into active site of 2F8A (ranked 181^st^) forming a hydrogen bond with ARG179. (**C**) ROCS overlap of nicotine with query molecule tiopronin and its TanimotoCombo rank with respect to all other drugs in the ROCS analyses. Panels A and B were created using the OE Chem Toolkit and Panel C was adapted from the ROCSReport utility program; both part of the OpenEye Software suite.

Nicotine docks reasonably well in 2F8A (181^st^) and 1GP1 (284^th^) however it falls outside the top 10% ranking in both models(Table II). In Fig. 4, nicotine is predicted to form hydrogen bonds with THR141 and with ASP142 in the 1GP1 crystal structure. In the 2F8A model it forms a hydrogen bond with the key amino acid residue ARG179. Compared to other molecules in the dataset, nicotine does have a similar shape to tiopronin (Fig. 4, Shape Tanimoto 0.829) and was ranked 94^th^ in the tiopronin ROCS model. The deleterious effects of cigarette smoking on lung function are well documented, however the extent to which nicotine itself may contribute to lung conditions is largely unknown [69]. These results suggest that nicotine may have the ability to alter GPx1 activity and is worthy of further investigation.

### Gabapentinoids

Pregabalin ranked 73^rd^ in 1GP1 and 112^th^ in 2F8A and ranked 39^th^ in ROCS comparison with tiopronin (Supplementary Tables 1 and 2). Similarly, gabapentin ranked 276^th^ in 1GP1 and 22^nd^ in 2F8A and ranked 26th in ROCS comparison with tiopronin (Supplementary Tables 1 and 2). In FAERS there were more than 200 reports for each of the outcomes; dyspnoea (highest number 2655), asthma, COPD, wheezing, cough, and productive cough associated with use of gabapentin (total number of ADE reports 232211). Similarly, more than 200 ADE reports that included dyspnoea (highest number 1185), COPD or cough were associated with pregabalin (total number of ADE reports 146229). Despite these findings, no statistically significant results were determined in the FAERS analysis for these drugs. The United States of America Food and Drug Administration, has previously warned of serious breathing difficulties in patients who take pregabalin or gabapentin particularly in people with respiratory risk factors [70]. Between January 1^st^ 2012 and October 26^th^ 2017 the FDA identified 49 reports of respiratory depression which mentioned the use of gabapentinoids [70]. Gabapentin has also previously been shown to reduce GPx activity in a rat brain demyelination model. GPx activity in the cortex decreased by 54.3% following administration of 300 mg/kg of gabapentin [71]. Gabapentinoids are becoming increasingly prescribed and along with this, their misuse and abuse above the normal therapeutic range is also increasing [72, 73]. Additional studies should be conducted to ascertain the risk of using gabapentinoids, particularly in people with compromised lung function.

### Previous *In Vitro* Investigations of Glutathione Peroxidase

Investigations of the effect of FDA approved drugs on erythrocyte GPx1 activity *in vitro* is scarce and often limited to animal studies. A literature search for *in vitro* data relating to the drugs listed in Table II revealed that when male rats were administered azathioprine 50 mg/kg this resulted in a 20% decrease in liver GPx levels compared to controls [74]. Mycophenolate mofetil (the prodrug of mycophenolic acid) at a dose of 100 mg/kg also reduced GPx activity by approximately 32% in a murine model [75]. Additionally, administration of voriconazole 50 mg/kg in murine models has also been shown to lower GPx activity and increase oxidative stress [76]. Additional molecules listed in Table II should be subjected to *in vitro* GPx1 analyses.

### Challenges and Opportunities

Despite the promising results shown by the multi-method approach detailed above, there are some challenging aspects which should be considered.

Dexlansoprazole appears to be associated with symptoms suggestive of COPD including cough and productive cough in FAERS (ROR 1.21 (95% CI 1.02–1.43) and ROR 2.82 (95% CI 1.01–2.02), respectively) and ranks well (111st) in the 2F8A docking model. Proton pump inhibitors may be associated with COPD exacerbations; however, this association may be representative of confounding by indication as gastro-oesophageal-reflux-disease is a common comorbidity of patients with COPD [77–79]. Similarly, despite furosemide ranking highly in the 1GP1 molecular model (144^th^), confounding by indication in FAERS data may be evident for furosemide since it can be used to treat symptoms of fluid overload in patients with COPD. Similar confounding might also explain the results for acetazolamide (FAERS COPD ADE signal ROR 2.71 (95% CI 1.50–4.89)), which can be used as a respiratory stimulant [80]. As confounding by indication is difficulty to control in FAERS analyses, an integrative approach to ADE detection and investigation is beneficial [81, 82].

The crystal structure of bovine GPx1 1GP1 was initially deposited in 1985 and is of poorer resolution (2.00 Å) compared to that of the human GPx1 2F8A model 1.50 Å [30]. No newer GPx1 crystal structures of the same origins are currently available [30]. Despite this, comparing bovine to human results was important to investigate any differences in the binding of drug molecules in the active site due to the mutation of the selenocysteine to glycine in the human model which had the potential to modulate the interactions that drugs had with this important amino acid residue in the active site. Importantly, in addition to the structure-based modelling analyses, ligand-based molecular modelling was also utilised to investigate and highlight important chemical features of molecules for GPx1 inhibition. FAERS is a spontaneous reporting system and generally reports are of adverse events that happen soon after medication administration [83]. COPD is an insidious condition and it is less likely that notifiers will report medication use and the development of a condition that occurs over time [84]. Moreover, it is difficult to search drug names in FAERS. Previously, researchers have mapped drug trade names to their common generic name using RxNorm, however in this analysis regular expressions were utilised, and they may have been too strict for this analysis [45, 83, 85].

Individually, both molecular modelling and pharmacoepidemiological methodologies have strengths in uncovering and investigating ADE signals [16, 86]. Computational molecular modelling provides an efficient and cost-effective method to rapidly determine which drugs may have favourable interactions in a target site of interest [87]. Pharmacoepidemiological analyses using spontaneous reporting systems such as FAERS provide real-world insight to ADE signals at a population level [88]. Together, an integrated multi-method approach to pharmacovigilance allows for improved signal detection by highlighting evidence for an ADE signal using several methods [15]. Moreover there is opportunity to advance drug safety by utilising complementary and integrative approaches, particularly to flag ADEs of drugs that may be new to market [18].

To our knowledge this study is the first to use an integrated multi-method approach to understand the effect that registered drugs may have on GPx1 and their propensity to cause ADEs. Additional analyses investigating the effect of registered medications on GPx1, and other oxidative stress pathways are required. Further optimisation of ADE signal detection, particularly due to GPx1 activity, should include *in vitro* analyses to determine implications of the ADE in humans at a biological level.

## Conclusion

This integrated molecular modelling and pharmacoepidemiological approach to adverse drug event signal detection and investigation has identified several drugs as potential inhibitors of GPx1. The Chronic Obstructive Disease adverse drug event signals associated with acetylsalicylic acid, atenolol, nicotine, gabapentinoids and dexlansoprazole may be of significant interest to clinicians.

To further explain and develop the models, *in vitro* analyses of relevant molecules would be of benefit. Moreover, additional longitudinal pharmacoepidemiological analyses may identify additional associations between these molecules and adverse drug events of interest.

## Supplementary Information

Below is the link to the electronic supplementary material.

Supplementary file1 (DOCX 62 KB)

## Data Availability

Data from FAERS are freely available. Data from DrugBank are freely available. The SAS code for FAERS analysis is available from the corresponding author on reasonable request.

## References

[1] Edwards IR, Aronson JK (2000). Adverse drug reactions: definitions, diagnosis, and management. Lancet..

[2] Pitts PJ, Louet HL, Moride Y, Conti RM (2016). 21st century pharmacovigilance: efforts, roles, and responsibilities. Lancet Oncol..

[3] Harmark L, van Grootheest AC (2008). Pharmacovigilance: methods, recent developments and future perspectives. Eur J Clin Pharmacol..

[4] Rodriguez LA, Wallander MA, Tolosa LB, Johansson S (2009). Chronic obstructive pulmonary disease in UK primary care: incidence and risk factors. COPD..

[5] Kelly TL, Ward M, Pratt NL, Ramsay E, Gillam M, Roughead EE (2022). The association between exacerbation of chronic obstructive pulmonary disease and timing of paracetamol use: a cohort study in elderly Australians. Respir Res..

[6] Dabo AJ, Ezegbunam W, Wyman AE, Moon J, Railwah C, Lora A (2020). Targeting c-Src Reverses Accelerated GPX-1 mRNA Decay in Chronic Obstructive Pulmonary Disease Airway Epithelial Cells. Am J Respir Cell Mol Biol..

[7] Vlahos R, Bozinovski S (2013). Glutathione peroxidase-1 as a novel therapeutic target for COPD. Redox Rep..

[8] World Health Organization: Chronic obstructive pulmonary disease (COPD). https://www.who.int/news-room/fact-sheets/detail/chronic-obstructive-pulmonary-disease-(copd) (2021). Accessed 16 February 2022.

[9] Rahman I, Adcock IM (2006). Oxidative stress and redox regulation of lung inflammation in COPD. Eur Respir J..

[10] Lubos E, Loscalzo J, Handy DE (2011). Glutathione peroxidase-1 in health and disease: from molecular mechanisms to therapeutic opportunities. Antioxid Redox Signal..

[11] Bentley AR, Emrani P, Cassano PA (2008). Genetic variation and gene expression in antioxidant related enzymes and risk of COPD: a systematic review. Thorax..

[12] Kluchova Z, Petrasova D, Joppa P, Dorkova Z, Tkacova R (2007). The association between oxidative stress and obstructive lung impairment in patients with COPD. Physiol Res..

[13] Santos MC, Oliveira AL, Viegas-Crespo AM, Vicente L, Barreiros A, Monteiro P (2004). Systemic markers of the redox balance in chronic obstructive pulmonary disease. Biomarkers..

[14] Shu M, Zai X, Zhang B, Wang R, Lin Z (2016). Hypothyroidism Side Effect in Patients Treated with Sunitinib or Sorafenib: Clinical and Structural Analyses. Plos One..

[15] Ho SS, McLachlan AJ, Chen TF, Hibbs DE, Fois RA (2015). Relationships Between Pharmacovigilance, Molecular, Structural, and Pathway Data: Revealing Mechanisms for Immune-Mediated Drug-Induced Liver Injury. CPT Pharmacometrics Syst Pharmacol..

[16] Schotland P, Bojunga N, Zien A, Trame MN, Lesko LJ (2016). Improving drug safety with a systems pharmacology approach. Eur J Pharm Sci..

[17] Tatonetti NP (2018). The Next Generation of Drug Safety Science: Coupling Detection, Corroboration, and Validation to Discover Novel Drug Effects and Drug-Drug Interactions. Clin Pharmacol Ther..

[18] Soldatos TG, Kim S, Schmidt S, Lesko LJ, Jackson DB (2022). Advancing drug safety science by integrating molecular knowledge with post-marketing adverse event reports. CPT Pharmacometrics Syst Pharmacol..

[19] U.S. Food and Drug Administration: FDA Adverse Event Reporting System (FAERS) Quarterly Data Extract Files. https://fis.fda.gov/extensions/FPD-QDE-FAERS/FPD-QDE-FAERS.html (2022). Accessed 1 February 2022.

[20] Wishart DS, Feunang YD, Guo AC, Lo EJ, Marcu A, Grant JR (2018). DrugBank 5.0: a major update to the DrugBank database for 2018. Nucleic Acids Res..

[21] Weininger D (1988). Smiles, a Chemical Language and Information-System. 1. Introduction to Methodology and Encoding Rules. J Chem Inf Comput Sci..

[22] Schulz R, Emmrich T, Lemmerhirt H, Leffler U, Sydow K, Hirt C (2012). Identification of a glutathione peroxidase inhibitor that reverses resistance to anticancer drugs in human B-cell lymphoma cell lines. Bioorg Med Chem Lett..

[23] Chaudiere J, Wilhelmsen EC, Tappel AL (1984). Mechanism of selenium-glutathione peroxidase and its inhibition by mercaptocarboxylic acids and other mercaptans. J Biol Chem..

[24] Wilde F, Lemmerhirt H, Emmrich T, Bednarski PJ, Link A (2014). Microwave-assisted synthesis and evaluation of acylhydrazones as potential inhibitors of bovine glutathione peroxidase. Mol Divers..

[25] Kumar KS, Weiss JF (1986). Inhibition of glutathione peroxidase and glutathione transferase in mouse liver by misonidazole. Biochem Pharmacol..

[26] Hall MD, Marshall TS, Kwit AD, Miller Jenkins LM, Dulcey AE, Madigan JP (2014). Inhibition of glutathione peroxidase mediates the collateral sensitivity of multidrug-resistant cells to tiopronin. J Biol Chem..

[27] Behnisch-Cornwell S, Laubenstein G, Bednarski PJ (2019). Studies of the inhibitory activities of tiopronin and mercaptosuccinic acid on glutathione peroxidase and their cytotoxic and antioxidant properties. Pharmazie..

[28] OpenEye Scientific: OpenEye Scientific. https://www.eyesopen.com/ (2018). Accessed 3rd July 2018 2018.

[29] Hawkins PC, Skillman AG, Warren GL, Ellingson BA, Stahl MT (2010). Conformer generation with OMEGA: algorithm and validation using high quality structures from the Protein Databank and Cambridge Structural Database. J Chem Inf Model..

[30] Epp O, Ladenstein R, Wendel A (1983). The refined structure of the selenoenzyme glutathione peroxidase at 0.2-nm resolution. Eur J Biochem..

[31] Kavanagh KL, Johansson, C., Smee, C., Gileadi, O., von Delft, F., Weigelt, J., Sundstrom, M., Edwards, A., Oppermann, U., : Crystal structure of the selenocysteine to glycine mutant of human glutathione peroxidase 1. https://www.wwpdb.org/pdb?id=pdb_00002f8a (2005). Accessed October 17 2021.

[32] Berman HM, Westbrook J, Feng Z, Gilliland G, Bhat TN, Weissig H (2000). The Protein Data Bank. Nucleic Acids Res..

[33] OEDOCKING. 3.4.0.2 ed. Santa Fe, NM: OpenEye Scientific Software, Inc., Santa Fen NM. http://www.eyesopen.com

[34] McGann M (2011). FRED pose prediction and virtual screening accuracy. J Chem Inf Model..

[35] Ali ST, Jahangir S, Karamat S, Fabian WM, Nawara K, Kona J (2010). Theoretical Study on the Redox Cycle of Bovine Glutathione Peroxidase GPx1: pKa Calculations, Docking, and Molecular Dynamics Simulations. J Chem Theory Comput..

[36] Ren B, Huang W Fau - Akesson B, Akesson B Fau - Ladenstein R, Ladenstein R. The crystal structure of seleno-glutathione peroxidase from human plasma at 2.9 A resolution. (0022–2836 (Print)). 10.1006/jmbi.1997.1005. 10.1006/jmbi.1997.1005 9180378

[37] McGann M (2012). FRED and HYBRID docking performance on standardized datasets. J Comput Aided Mol Des..

[38] Kelley BP, Brown SP, Warren GL, Muchmore SW (2015). POSIT: Flexible Shape-Guided Docking For Pose Prediction. J Chem Inf Model..

[39] UniProt C (2021). UniProt: the universal protein knowledgebase in 2021. Nucleic Acids Res..

[40] Ramirez D, Caballero J (2018). Is It Reliable to Take the Molecular Docking Top Scoring Position as the Best Solution without Considering Available Structural Data?. Mol..

[41] Warren GL, Andrews CW, Capelli AM, Clarke B, LaLonde J, Lambert MH (2006). A critical assessment of docking programs and scoring functions. J Med Chem..

[42] Azam SS, Abbasi SW (2013). Molecular docking studies for the identification of novel melatoninergic inhibitors for acetylserotonin-O-methyltransferase using different docking routines. Theor Biol Med Model..

[43] Eisenhaber F. Discovering Biomolecular Mechanisms with Computational Biology. Molecular biology intelligence unit (Unnumbered). Georgetown, Tex. : New York: Landes Bioscience/Eurekah.com ; Springer Science+Business Media; 2006. 10.1007/0-387-36747-0.

[44] Hawkins PC, Skillman AG, Nicholls A (2007). Comparison of shape-matching and docking as virtual screening tools. J Med Chem..

[45] Banda JM, Evans L, Vanguri RS, Tatonetti NP, Ryan PB, Shah NH (2016). A curated and standardized adverse drug event resource to accelerate drug safety research. Sci Data..

[46] Toelle BG, Xuan W, Bird TE, Abramson MJ, Atkinson DN, Burton DL (2013). Respiratory symptoms and illness in older Australians: the Burden of Obstructive Lung Disease (BOLD) study. Med J Aust..

[47] Liu Y, Pleasants RA, Croft JB, Wheaton AG, Heidari K, Malarcher AM (2015). Smoking duration, respiratory symptoms, and COPD in adults aged >/=45 years with a smoking history. Int J Chron Obstruct Pulmon Dis..

[48] Truven Health Analytics I, Micromedex I. Micromedex. Micromedex gateway: Chicago, Ill.. : Truven Health Analytics; 2013. https://www.micromedexsolutions.com/home/dispatch.

[49] SAS Statistical Software: SAS Help Center: Using Perl Regular Expressions in the DATA Step. https://documentation.sas.com/doc/en/pgmsascdc/9.4_3.2/lefunctionsref/p1vz3ljudbd756n19502acxazevk.htm (2022). Accessed 1 February 2022 2022.

[50] Aw I, Pratt NL, Kalisch LM, Roughead EE (2014). Comparing time to adverse drug reaction signals in a spontaneous reporting database and a claims database: a case study of rofecoxib-induced myocardial infarction and rosiglitazone-induced heart failure signals in Australia. Drug Saf..

[51] Muñoz MA, Tonning JM, Brinker AD, Delaney JAC, Gatti JC, Avigan M (2016). Data Mining of the US FDA’s Adverse Events Reporting System Database to Evaluate Drug-Drug Interactions Associated with Statin-Induced Rhabdomyolysis. Pharma Med..

[52] Szumilas M (2010). Explaining odds ratios. J Can Acad Child Adolesc Psych..

[53] Rothman KJ, Lanes S, Sacks ST (2004). The reporting odds ratio and its advantages over the proportional reporting ratio. Pharmacoepidemiol Drug Saf..

[54] Ang PS, Chen Z, Chan CL, Tai BC (2016). Data mining spontaneous adverse drug event reports for safety signals in Singapore - a comparison of three different disproportionality measures. Expert Opin Drug Saf..

[55] Schmidt RL, Simonovic M (2012). Synthesis and decoding of selenocysteine and human health. Croat Med J..

[56] Ursini F, Maiorino M, Lennarz WJ, Lane MD (2004). Glutathione Peroxidases. Encyclopedia of Biological Chemistry.

[57] Dimastrogiovanni D, Anselmi M, Miele AE, Boumis G, Petersson L, Angelucci F (2010). Combining crystallography and molecular dynamics: the case of Schistosoma mansoni phospholipid glutathione peroxidase. Proteins..

[58] Jaffe IA (1986). Adverse effects profile of sulfhydryl compounds in man. Am J Med..

[59] Dorow P, Bethge H, Tonnesmann U (1986). Effects of single oral doses of bisoprolol and atenolol on airway function in nonasthmatic chronic obstructive lung disease and angina pectoris. Eur J Clin Pharmacol..

[60] Gulea C, Zakeri R, Alderman V, Morgan A, Ross J, Quint JK (2021). Beta-blocker therapy in patients with COPD: a systematic literature review and meta-analysis with multiple treatment comparison. Respir Res..

[61] Baker JG, Wilcox RG (2017). beta-Blockers, heart disease and COPD: current controversies and uncertainties. Thorax..

[62] Dorow P, Thalhofer S, Bethge H, Disselhoff G, Wagner G (1990). Long-term treatment of angina pectoris with bisoprolol or atenolol in patients with chronic obstructive bronchitis: a randomized, double-blind crossover study. J Cardiovasc Pharmacol..

[63] Kacprzak D, Pawliczak R (2015). Does aspirin-induced oxidative stress cause asthma exacerbation?. Arch Med Sci..

[64] Varghese M, Lockey RF (2008). Aspirin-exacerbated asthma. Allergy Asthma Clin Immunol..

[65] Hassan AM (2003). Glutathione peroxidase activity in blood cells from aspirin-induced asthma patients. Ann Clin Biochem..

[66] Vally H, Taylor ML, Thompson PJ (2002). The prevalence of aspirin intolerant asthma (AIA) in Australian asthmatic patients. Thorax..

[67] Huang KY, Tseng PT, Wu YC, Tu YK, Stubbs B, Su KP (2021). Do beta-adrenergic blocking agents increase asthma exacerbation? A network meta-analysis of randomized controlled trials. Sci Rep..

[68] Morales DR, Lipworth BJ, Donnan PT, Jackson C, Guthrie B (2017). Respiratory effect of beta-blockers in people with asthma and cardiovascular disease: population-based nested case control study. BMC Med..

[69] Giuca MR, Giuggioli E, Metelli MR, Pasini M, Iezzi G, D'Ercole S (2010). Effects of cigarette smoke on salivary superoxide dismutase and glutathione peroxidase activity. J Biol Regul Homeost Agents..

[70] U.S. Food and Drug Administration: FDA warns about serious breathing FDA warns about serious breathing problems with seizure and nerve pain medicines gabapentin (Neurontin, Gralise, Horizant) and pregabalin (Lyrica, Lyrica CR) When used with CNS depressants or in patients with lung problems. https://www.fda.gov/drugs/drug-safety-and-availability/fda-warns-about-serious-breathing-problems-seizure-and-nerve-pain-medicines-gabapentin-neurontin (2019). Accessed 15 December 2020.

[71] Abdel-Salam OM, Khadrawy YA, Mohammed NA, Youness ER (2012). The effect of gabapentin on oxidative stress in a model of toxic demyelination in rat brain. J Basic Clin Physiol Pharmacol..

[72] Evoy KE, Covvey JR, Peckham AM, Ochs L, Hultgren KE (2019). Reports of gabapentin and pregabalin abuse, misuse, dependence, or overdose: An analysis of the Food And Drug Administration Adverse Events Reporting System (FAERS). Res Social Adm Pharm..

[73] Cairns R, Schaffer AL, Ryan N, Pearson SA, Buckley NA (2019). Rising pregabalin use and misuse in Australia: trends in utilization and intentional poisonings. Addict..

[74] El-Beshbishy HA, Tork OM, El-Bab MF, Autifi MA (2011). Antioxidant and antiapoptotic effects of green tea polyphenols against azathioprine-induced liver injury in rats. Pathophysiol..

[75] Dalmarco EM, Budni P, Parisotto EB, Wilhelm Filho D, Frode TS (2009). Antioxidant effects of mycophenolate mofetil in a murine pleurisy model. Transpl Immunol..

[76] Wu SL, Wei TY, Lin SW, Su KY, Kuo CH (2019). Metabolomics Investigation of Voriconazole-Induced Hepatotoxicity in Mice. Chem Res Toxicol..

[77] Janetzki JL, Sykes MJ, Ward MB, Pratt NL (2021). Proton pump inhibitors may contribute to progression or development of chronic obstructive pulmonary disease-A sequence symmetry analysis approach. J Clin Pharm Ther..

[78] Tan J, Li L, Huang X, Yang C, Liang X, Zhao Y (2020). Associations between gastro-oesophageal reflux disease and a range of diseases: an umbrella review of systematic reviews and meta-analyses. BMJ Open..

[79] Kikuchi S, Naoki Y, Tajiri T, Watanabe N (2018). Proton pump inhibitors for chronic obstructive pulmonary disease. Cochrane Database System Rev..

[80] Faisy C, Meziani F, Planquette B, Clavel M, Gacouin A, Bornstain C (2016). Effect of Acetazolamide vs Placebo on Duration of Invasive Mechanical Ventilation Among Patients With Chronic Obstructive Pulmonary Disease: A Randomized Clinical Trial. JAMA..

[81] Prada-Ramallal G, Takkouche B, Figueiras A (2019). Bias in pharmacoepidemiologic studies using secondary health care databases: a scoping review. BMC Med Res Methodol..

[82] Signorello LB, McLaughlin JK, Lipworth L, Friis S, Sorensen HT, Blot WJ (2002). Confounding by indication in epidemiologic studies of commonly used analgesics. Am J Ther..

[83] Yu Y, Ruddy KJ, Hong N, Tsuji S, Wen A, Shah ND (2019). ADEpedia-on-OHDSI: A next generation pharmacovigilance signal detection platform using the OHDSI common data model. J Biomed Inform..

[84] Yang I, Brown J, George J, Jenkins S, McDonald C, McDonald V, *et al.* The COPD-X Plan: Australian and New Zealand Guidelines for the management of Chronic Obstructive Pulmonary Disease 2019. 2019. 10.5694/mja17.00686 29129177

[85] Davidson L, Boland MR. Comparative analysis and evaluation of State-of-the-Art medication mapping tools to transform a local medication terminology to RxNorm. AMIA Jt Summits Transl Sci Proc. 2020;2020:126–35. PMC7233099 32477631

[86] Vilar S, Ryan PB, Madigan D, Stang PE, Schuemie MJ, Friedman C (2014). Similarity-based modeling applied to signal detection in pharmacovigilance. CPT Pharmacometrics Syst Pharmacol..

[87] Liu R, Zhang P (2019). Towards early detection of adverse drug reactions: combining pre-clinical drug structures and post-market safety reports. BMC Med Inform Decis Mak..

[88] Sonawane KB, Cheng N, Hansen RA (2018). Serious Adverse Drug Events Reported to the FDA: Analysis of the FDA Adverse Event Reporting System 2006–2014 Database. J Manag Care Spec Pharm..

