# Supplementary material for: Application of an Integrative Drug Safety Model for Detection of Adverse Drug Events Associated With Inhibition of Glutathione Peroxidase 1 in Chronic Obstructive Pulmonary Disease
Source: Pharm Res. 2023 May 12;40(6):1553–68. doi: 10.1007/s11095-023-03516-x (PMC10338407; doi:10.1007/s11095-023-03516-x)
Supplement: Supplementary file 1 — Supplementary file1 (DOCX 62 KB) [file 11095_2023_3516_MOESM1_ESM.docx]

**Supplementary information**

**Supplementary Table 1: Docking ranks of drug molecules into active site of GPX1 PDB code 1GP1 (bovine) and 2F8A (human)**

* denotes a known GPx1 inhibitor

|  | **1GP1** | | **2F8A** | |
| --- | --- | --- | --- | --- |
| **Docking rank** | **Molecule Name** | **FRED Chemgauss4 score**  **(docking score)** | **Molecule Name** | **FRED Chemgauss4 score (docking score)** |
| 1 | Phenformin | -8.795 | Carglumic acid | -6.913 |
| 2 | L-Tryptophan | -8.575 | Amifostine | -6.840 |
| 3 | Inosine pranobex | -8.540 | Triamterene | -6.734 |
| 4 | Mepiprazole | -8.446 | Tartaric acid | -6.370 |
| 5 | N-acetyltyrosine | -8.295 | Azelaic acid | -6.210 |
| 6 | Pidotimod | -8.293 | Decitabine | -6.007 |
| 7 | Chlorphenamine | -8.239 | Ceftibuten | -5.841 |
| 8 | Brompheniramine | -8.185 | Acetylcysteine | -5.729 |
| 9 | Methyldopa | -8.170 | Inosine | -5.593 |
| 10 | Levodopa | -8.127 | Mercaptosuccinate* | -5.550 |
| 11 | Chlorphenesin | -7.959 | Levodopa | -5.407 |
| 12 | Enoximone | -7.935 | L-Lysine | -5.363 |
| 13 | Levonordefrin | -7.883 | Adenine | -5.287 |
| 14 | L-Lysine | -7.839 | Aminosalicylic acid | -5.174 |
| 15 | Tioguanine | -7.815 | Ceftizoxime | -5.168 |
| 16 | Amifostine | -7.792 | Allopurinol | -5.162 |
| 17 | Pyridoxine | -7.754 | Calcium threonate | -5.156 |
| 18 | Pindolol | -7.563 | Nitrofural | -5.117 |
| 19 | Salsalate | -7.496 | Aminolevulinic acid | -5.086 |
| 20 | Bufexamac | -7.427 | Trapidil | -5.070 |
| 21 | Isoprenaline | -7.396 | Didanosine | -5.067 |
| 22 | Ganciclovir | -7.381 | Gabapentin | -5.043 |
| 23 | Telbivudine | -7.368 | Aminohippuric acid | -5.032 |
| 24 | Fenbufen | -7.350 | Mercaptopurine | -5.018 |
| 25 | Epinephrine | -7.348 | Lamivudine | -4.994 |
| 26 | Norepinephrine | -7.320 | Tioguanine | -4.948 |
| 27 | Oxitriptan | -7.314 | Methionine | -4.930 |
| 28 | Hydroxyamphetamine | -7.303 | Demethyl tiopronin* | -4.928 |
| 29 | 3eC7* | -7.292 | Nicorandil | -4.915 |
| 30 | Propylthiouracil | -7.262 | L-Isoleucine | -4.908 |
| 31 | Carprofen | -7.175 | Salicylic acid | -4.900 |
| 32 | Alosetron | -7.173 | Zalcitabine | -4.895 |
| 33 | Phenylephrine | -7.142 | Salsalate | -4.888 |
| 34 | Dopamine | -7.133 | D-Methionine | -4.859 |
| 35 | Pentostatin | -7.112 | Racemethionine | -4.859 |
| 36 | Compound 3* | -7.101 | Phenylacetic acid | -4.859 |
| 37 | 8-chlorotheophylline | -7.094 | Calcium saccharate | -4.856 |
| 38 | Dexibuprofen | -7.074 | Tiopronin* | -4.831 |
| 39 | Ibuprofen | -7.074 | Gluconate | -4.815 |
| 40 | Melatonin | -7.056 | Acyclovir | -4.786 |
| 41 | Labetalol | -7.046 | Valproic acid | -4.771 |
| 42 | Levosalbutamol | -7.024 | Epirizole | -4.745 |
| 43 | Salbutamol | -7.024 | Pirbuterol | -4.741 |
| 44 | Niflumic Acid | -7.009 | Ribavirin | -4.738 |
| 45 | Mefenamic acid | -7.000 | Pantothenic acid | -4.735 |
| 46 | Eugenol | -6.971 | Methyldopa | -4.699 |
| 47 | Bethanidine | -6.952 | Gimeracil | -4.686 |
| 48 | Isoetarine | -6.944 | Adenosine | -4.655 |
| 49 | Ramosetron | -6.931 | Isoniazid | -4.646 |
| 50 | Mephenesin | -6.891 | Temozolomide | -4.624 |
| 51 | 3aC7* | -6.883 | Penciclovir | -4.624 |
| 52 | Mercaptopurine | -6.868 | Epinephrine | -4.605 |
| 53 | Tofacitinib | -6.857 | Phenacemide | -4.604 |
| 54 | Atenolol | -6.840 | N-acetyltyrosine | -4.580 |
| 55 | Bromotheophylline | -6.824 | Nitroxoline | -4.565 |
| 56 | Rilpivirine | -6.801 | Ethosuximide | -4.557 |
| 57 | Polaprezinc | -6.785 | Mesalazine | -4.542 |
| 58 | Enprofylline | -6.752 | Captopril | -4.509 |
| 59 | Tripelennamine | -6.744 | Levonordefrin | -4.501 |
| 60 | Furazolidone | -6.727 | Emtricitabine | -4.476 |
| 61 | Penciclovir | -6.723 | Ganciclovir | -4.474 |
| 62 | Carglumic Acid | -6.722 | Polaprezinc | -4.447 |
| 63 | Tipiracil | -6.718 | Cilastatin | -4.429 |
| 64 | Esculin | -6.714 | Acamprosate | -4.420 |
| 65 | Rizatriptan | -6.696 | Cinoxacin | -4.412 |
| 66 | Lornoxicam | -6.696 | Stepronin | -4.403 |
| 67 | Hexylresorcinol | -6.689 | Acetylsalicylic acid | -4.391 |
| 68 | Guaifenesin | -6.680 | L-Carnitine | -4.390 |
| 69 | Dioxybenzone | -6.655 | Carbenicillin | -4.363 |
| 70 | Pheniramine | -6.650 | Fluciclovine (18F) | -4.352 |
| 71 | Tranexamic Acid | -6.630 | Furazolidone | -4.351 |
| 72 | Monoctanoin | -6.628 | Ataluren | -4.339 |
| 73 | Pregabalin | -6.610 | Naproxen | -4.324 |
| 74 | L-Tyrosine | -6.586 | Nitrofurantoin | -4.299 |
| 75 | Metaraminol | -6.579 | Cytarabine | -4.296 |
| 76 | Chloropyramine | -6.564 | Ticarcillin | -4.284 |
| 77 | Floxuridine | -6.552 | Pemirolast | -4.281 |
| 78 | Temozolomide | -6.543 | Aminobenzoic acid | -4.274 |
| 79 | Tolcapone | -6.536 | Pyrimethamine | -4.256 |
| 80 | Midodrine | -6.506 | Phenylbutyric acid | -4.247 |
| 81 | Ruxolitinib | -6.487 | Voriconazole | -4.240 |
| 82 | Alclofenac | -6.486 | Sulfacytine | -4.232 |
| 83 | Fenoterol | -6.478 | L-Leucine | -4.232 |
| 84 | Uracil mustard | -6.477 | L-Phenylalanine | -4.232 |
| 85 | Acyclovir | -6.470 | Nalidixic acid | -4.231 |
| 86 | Toloxatone | -6.466 | Loxoprofen | -4.211 |
| 87 | Phenyl aminosalicylate | -6.464 | Allantoin | -4.203 |
| 88 | Arbutin | -6.462 | Tranexamic acid | -4.198 |
| 89 | Adenine | -6.461 | Vitamin C | -4.191 |
| 90 | Azelaic Acid | -6.456 | Tizanidine | -4.160 |
| 91 | Fenoprofen | -6.448 | L-Tyrosine | -4.158 |
| 92 | Tolfenamic Acid | -6.440 | Methyl nicotinate | -4.153 |
| 93 | Gimeracil | -6.424 | Alclofenac | -4.140 |
| 94 | Aminosalicylic Acid | -6.421 | Mercaptovaline* | -4.139 |
| 95 | Iobenguane sulfate I-123 | -6.414 | Cefprozil | -4.135 |
| 96 | Doxylamine | -6.400 | Diflunisal | -4.118 |
| 97 | Cimetidine | -6.399 | Gemcitabine | -4.110 |
| 98 | Meprobamate | -6.395 | Penicillamine | -4.100 |
| 99 | Iobenguane | -6.389 | Lamotrigine | -4.099 |
| 100 | Dobutamine | -6.381 | Inosine pranobex | -4.097 |
| 101 | Mycophenolic acid | -6.369 | Kinetin | -4.094 |
| 102 | Vitamin C | -6.362 | 3eC7* | -4.083 |
| 103 | Clonixin | -6.362 | Anthralin | -4.075 |
| 104 | Nitrofural | -6.356 | Diiodohydroxyquinoline | -4.070 |
| 105 | Mitiglinide | -6.348 | Clioquinol | -4.062 |
| 106 | Metformin | -6.340 | Cefixime | -4.057 |
| 107 | Ataluren | -6.324 | Metyrapone | -4.037 |
| 108 | Baricitinib | -6.322 | L-Tryptophan | -4.037 |
| 109 | Hexaminolevulinate | -6.319 | Glycodiazine | -4.037 |
| 110 | Dextroamphetamine | -6.319 | Mefenamic acid | -4.000 |
| 111 | Amphetamine | -6.319 | Dexlansoprazole | -3.983 |
| 112 | Brivaracetam | -6.314 | Pregabalin | -3.972 |
| 113 | Phenacemide | -6.307 | Acetylcarnitine | -3.963 |
| 114 | Levmetamfetamine | -6.307 | Oxyquinoline | -3.959 |
| 115 | Decitabine | -6.294 | Compound 3* | -3.944 |
| 116 | Theophylline | -6.290 | 3aC7* | -3.942 |
| 117 | Fenspiride | -6.251 | Fluorouracil | -3.931 |
| 118 | Debrisoquin | -6.242 | Sulfamethazine | -3.919 |
| 119 | Clioquinol | -6.237 | Imipenem | -3.915 |
| 120 | Oxymetazoline | -6.214 | Chloroxine | -3.899 |
| 121 | Calcium saccharate | -6.212 | Suprofen | -3.882 |
| 122 | Zalcitabine | -6.206 | Protionamide | -3.874 |
| 123 | Carteolol | -6.197 | Sulfamerazine | -3.872 |
| 124 | Vanillyl butyl ether | -6.173 | Norepinephrine | -3.864 |
| 125 | Terbutaline | -6.169 | Acetazolamide | -3.860 |
| 126 | Ribavirin | -6.166 | Azathioprine | -3.857 |
| 127 | Oxyquinoline | -6.161 | Ciclopirox | -3.843 |
| 128 | Salicylamide | -6.161 | Fenbufen | -3.840 |
| 129 | Kinetin | -6.157 | Oxitriptan | -3.834 |
| 130 | Isosorbide Mononitrate | -6.156 | Riboflavin | -3.808 |
| 131 | Nitrofurantoin | -6.147 | Sulfadiazine | -3.801 |
| 132 | Dantrolene | -6.143 | Cefdinir | -3.800 |
| 133 | Metaxalone | -6.117 | 3aC9* | -3.793 |
| 134 | Epirizole | -6.116 | Brimonidine | -3.792 |
| 135 | Metyrosine | -6.114 | Cefuroxime | -3.770 |
| 136 | Sulisobenzone | -6.103 | Bendamustine | -3.766 |
| 137 | Aminolevulinic acid | -6.101 | Brivaracetam | -3.762 |
| 138 | Pentamidine | -6.099 | Dexpanthenol | -3.737 |
| 139 | Amylmetacresol | -6.087 | Tiaprofenic acid | -3.734 |
| 140 | Benznidazole | -6.073 | Pentostatin | -3.727 |
| 141 | Biotin | -6.073 | Pidotimod | -3.720 |
| 142 | Fluorouracil | -6.071 | Danthron | -3.714 |
| 143 | Imipenem | -6.070 | Biotin | -3.707 |
| 144 | Furosemide | -6.065 | Isoflurophate | -3.705 |
| 145 | Ethionamide | -6.062 | Dexibuprofen | -3.701 |
| 146 | Isometheptene | -6.052 | Ibuprofen | -3.701 |
| 147 | Chloroxine | -6.030 | Sulfameter | -3.697 |
| 148 | Baclofen | -6.020 | Clonixin | -3.689 |
| 149 | Gluconate | -6.015 | Diatrizoate | -3.685 |
| 150 | Loxoprofen | -6.009 | Baclofen | -3.672 |

**Supplementary Table 2: ROCS ranks of drug molecules compared to known inhibitor tiopronin**

* denotes a known GPx1 inhibitor

| **Molecule** | **Tanimoto Combo** | **Shape Tanimoto** | **Color Tanimoto** | **Rank** |
| --- | --- | --- | --- | --- |
| Tiopronin* | 2.00 | 1.00 | 1.00 | 1 |
| Demethyl tiopronin | 1.92 | 0.92 | 1.00 | 2 |
| Acetylcysteine | 1.65 | 0.71 | 0.94 | 3 |
| Aminolevulinicacid | 1.49 | 0.92 | 0.57 | 4 |
| Mercaptosuccinate* | 1.38 | 0.82 | 0.56 | 5 |
| Phenylacetic acid | 1.35 | 0.85 | 0.50 | 6 |
| L-Carnitine | 1.33 | 0.81 | 0.52 | 7 |
| Aminohippuric acid | 1.32 | 0.70 | 0.63 | 8 |
| Stepronin | 1.32 | 0.60 | 0.71 | 9 |
| Phenylbutyric acid | 1.26 | 0.76 | 0.50 | 10 |
| Methionine | 1.22 | 0.85 | 0.37 | 11 |
| Acetylcarnitine | 1.21 | 0.56 | 0.65 | 12 |
| Tranexamic acid | 1.20 | 0.82 | 0.38 | 13 |
| Tartaric acid | 1.19 | 0.81 | 0.38 | 14 |
| Protionamide | 1.19 | 0.82 | 0.37 | 15 |
| D-Methionine | 1.19 | 0.81 | 0.38 | 16 |
| Racemethionine | 1.19 | 0.81 | 0.38 | 17 |
| L-Lysine | 1.18 | 0.85 | 0.33 | 18 |
| Methyl aminolevulinate | 1.18 | 0.90 | 0.29 | 19 |
| Acamprosate | 1.15 | 0.82 | 0.33 | 20 |
| Calcium threonate | 1.14 | 0.70 | 0.44 | 21 |
| L-Phenylalanine | 1.13 | 0.76 | 0.38 | 22 |
| Ethionamide | 1.13 | 0.85 | 0.28 | 23 |
| L-Isoleucine | 1.13 | 0.79 | 0.34 | 24 |
| Methyl nicotinate | 1.13 | 0.81 | 0.32 | 25 |
| Gabapentin | 1.10 | 0.73 | 0.37 | 26 |
| Pantothenic acid | 1.10 | 0.69 | 0.41 | 27 |
| Gluconate | 1.10 | 0.85 | 0.25 | 28 |
| Carglumic acid | 1.10 | 0.57 | 0.53 | 29 |
| Acetylsalicylic acid | 1.08 | 0.53 | 0.55 | 30 |
| Salicylic acid | 1.08 | 0.71 | 0.37 | 31 |
| Piracetam | 1.07 | 0.79 | 0.28 | 32 |
| Valproic acid | 1.07 | 0.70 | 0.38 | 33 |
| Tolfenamic acid | 1.07 | 0.56 | 0.52 | 34 |
| Mefenamic acid | 1.07 | 0.55 | 0.52 | 35 |
| Isosorbide mononitrate | 1.07 | 0.81 | 0.25 | 36 |
| Acetylcholine | 1.06 | 0.89 | 0.17 | 37 |
| Alclofenac | 1.06 | 0.68 | 0.38 | 38 |
| Pregabalin | 1.06 | 0.73 | 0.33 | 39 |
| Calcium saccharate | 1.05 | 0.77 | 0.28 | 40 |
| L-Leucine | 1.04 | 0.67 | 0.37 | 41 |
| Ibuprofen | 1.04 | 0.67 | 0.37 | 42 |
| Dexibuprofen | 1.04 | 0.67 | 0.37 | 43 |
| Aminobenzoic acid | 1.04 | 0.65 | 0.39 | 44 |
| Carbachol | 1.04 | 0.90 | 0.14 | 45 |
| Mesalazine | 1.04 | 0.74 | 0.29 | 46 |
| Gemfibrozil | 1.03 | 0.53 | 0.51 | 47 |
| Clonixin | 1.03 | 0.50 | 0.54 | 48 |
| Meclofenamic acid | 1.02 | 0.51 | 0.52 | 49 |
| Fluciclovine (18F) | 1.02 | 0.65 | 0.38 | 50 |
| Penicillamine | 1.02 | 0.45 | 0.57 | 51 |
| Mercaptovaline* | 1.00 | 0.63 | 0.38 | 52 |
| Azelaic acid | 1.00 | 0.63 | 0.38 | 53 |
| Captopril | 1.00 | 0.64 | 0.37 | 54 |
| L-Tyrosine | 1.00 | 0.70 | 0.30 | 55 |
| Isoflurophate | 1.00 | 0.86 | 0.14 | 56 |
| Chlorambucil | 1.00 | 0.50 | 0.50 | 57 |
| Phenylpropanolamine | 0.99 | 0.88 | 0.11 | 58 |
| Pseudoephedrine | 0.99 | 0.88 | 0.11 | 59 |
| Baclofen | 0.99 | 0.61 | 0.37 | 60 |
| Triflusal | 0.98 | 0.43 | 0.55 | 61 |
| Acetazolamide | 0.98 | 0.70 | 0.29 | 62 |
| Eugenol | 0.98 | 0.74 | 0.23 | 63 |
| Phenoxyethanol | 0.98 | 0.85 | 0.13 | 64 |
| Mephenesin | 0.98 | 0.77 | 0.21 | 65 |
| Niflumic acid | 0.98 | 0.44 | 0.54 | 66 |
| Dexpanthenol | 0.98 | 0.74 | 0.24 | 67 |
| Sulfanilamide | 0.98 | 0.76 | 0.21 | 68 |
| Phenylephrine | 0.97 | 0.79 | 0.18 | 69 |
| Mafenide | 0.97 | 0.76 | 0.21 | 70 |
| Dopamine | 0.97 | 0.79 | 0.19 | 71 |
| Aminosalicylic acid | 0.97 | 0.65 | 0.32 | 72 |
| Isoniazid | 0.97 | 0.73 | 0.24 | 73 |
| Glycol salicylate | 0.96 | 0.74 | 0.22 | 74 |
| Metaraminol | 0.96 | 0.78 | 0.18 | 75 |
| Betahistine | 0.96 | 0.84 | 0.13 | 76 |
| Guaifenesin | 0.96 | 0.69 | 0.27 | 77 |
| Nitroxoline | 0.96 | 0.62 | 0.34 | 78 |
| Metyrosine | 0.96 | 0.66 | 0.30 | 79 |
| L-Tryptophan | 0.96 | 0.65 | 0.30 | 80 |
| Nicorandil | 0.95 | 0.62 | 0.34 | 81 |
| Levetiracetam | 0.95 | 0.71 | 0.25 | 82 |
| Oxaprozin | 0.95 | 0.46 | 0.50 | 83 |
| Naproxen | 0.95 | 0.62 | 0.33 | 84 |
| Terpin hydrate | 0.95 | 0.74 | 0.21 | 85 |
| Amifostine | 0.95 | 0.69 | 0.26 | 86 |
| Fluorouracil | 0.95 | 0.60 | 0.35 | 87 |
| Carbimazole | 0.95 | 0.82 | 0.13 | 88 |
| Flurbiprofen | 0.95 | 0.57 | 0.38 | 89 |
| Pemirolast | 0.95 | 0.56 | 0.38 | 90 |
| Diethylcarbamazine | 0.95 | 0.82 | 0.12 | 91 |
| Chlorphenesin | 0.95 | 0.74 | 0.20 | 92 |
| Pyridostigmine | 0.94 | 0.78 | 0.17 | 93 |
| Nicotine | 0.94 | 0.83 | 0.12 | 94 |
| Misonidazole* | 0.94 | 0.73 | 0.21 | 95 |
| Fenoprofen | 0.94 | 0.46 | 0.48 | 96 |
| Benzocaine | 0.94 | 0.77 | 0.16 | 97 |
| Methazolamide | 0.94 | 0.74 | 0.19 | 98 |
| Zonisamide | 0.94 | 0.72 | 0.22 | 99 |
| Diethyltoluamide | 0.93 | 0.76 | 0.17 | 100 |
| Malathion | 0.93 | 0.54 | 0.39 | 101 |
| Pidotimod | 0.93 | 0.63 | 0.30 | 102 |
| Pralidoxime | 0.93 | 0.73 | 0.20 | 103 |
| Bronopol | 0.93 | 0.61 | 0.32 | 104 |
| Edrophonium | 0.92 | 0.78 | 0.14 | 105 |
| Menthol | 0.92 | 0.80 | 0.13 | 106 |
| Ethosuximide | 0.92 | 0.68 | 0.25 | 107 |
| Levonordefrin | 0.92 | 0.72 | 0.21 | 108 |
| Ephedrine | 0.92 | 0.81 | 0.11 | 109 |
| Methyl salicylate | 0.92 | 0.80 | 0.13 | 110 |
| Phenacemide | 0.92 | 0.56 | 0.36 | 111 |
| Diflunisal | 0.92 | 0.59 | 0.33 | 112 |
| Norepinephrine | 0.92 | 0.74 | 0.18 | 113 |
| Levodopa | 0.92 | 0.66 | 0.25 | 114 |
| Aceclofenac | 0.92 | 0.42 | 0.50 | 115 |
| DL-Methylephedrine | 0.91 | 0.80 | 0.11 | 116 |
| Trapidil | 0.91 | 0.66 | 0.25 | 117 |
| Metamizole | 0.91 | 0.51 | 0.41 | 118 |
| Uracil mustard | 0.91 | 0.56 | 0.35 | 119 |
| Methacholine | 0.91 | 0.74 | 0.17 | 120 |
| Allantoin | 0.91 | 0.68 | 0.24 | 121 |
| Isometheptene | 0.91 | 0.91 | 0.00 | 122 |
| Suprofen | 0.91 | 0.58 | 0.33 | 123 |
| Diclofenac | 0.91 | 0.47 | 0.44 | 124 |
| Tinidazole | 0.91 | 0.58 | 0.33 | 125 |
| N-acetyltyrosine | 0.90 | 0.60 | 0.30 | 126 |
| Gimeracil | 0.90 | 0.62 | 0.29 | 127 |
| Loxoprofen | 0.90 | 0.57 | 0.33 | 128 |
| Paramethadione | 0.90 | 0.62 | 0.28 | 129 |
| Tocainide | 0.90 | 0.65 | 0.25 | 130 |
| Ciprofibrate | 0.90 | 0.53 | 0.37 | 131 |
| Mitiglinide | 0.90 | 0.41 | 0.49 | 132 |
| Epinephrine | 0.90 | 0.74 | 0.16 | 133 |
| Monoctanoin | 0.90 | 0.63 | 0.26 | 134 |
| Nalidixic acid | 0.89 | 0.54 | 0.35 | 135 |
| Vitamin C | 0.89 | 0.68 | 0.21 | 136 |
| Bethanechol | 0.89 | 0.75 | 0.14 | 137 |
| Tiaprofenic acid | 0.89 | 0.56 | 0.33 | 138 |
| Dichlorobenzyl alcohol | 0.89 | 0.75 | 0.14 | 139 |
| Kinetin | 0.89 | 0.64 | 0.25 | 140 |
| Butamben | 0.89 | 0.74 | 0.15 | 141 |
| Sodium lauryl sulfate | 0.88 | 0.50 | 0.38 | 142 |
| Ethchlorvynol | 0.88 | 0.76 | 0.13 | 143 |
| Fenproporex | 0.88 | 0.61 | 0.27 | 144 |
| Tegafur | 0.88 | 0.64 | 0.24 | 145 |
| Diethylpropion | 0.88 | 0.75 | 0.13 | 146 |
| Methyldopa | 0.88 | 0.63 | 0.25 | 147 |
| Metformin | 0.88 | 0.78 | 0.10 | 148 |
| Hydroxyamphetamine | 0.88 | 0.77 | 0.11 | 149 |
| Betiatide | 0.88 | 0.38 | 0.50 | 150 |
